# The relation between earthquake stress coping strategies and post-earthquake trauma level

**DOI:** 10.1097/MD.0000000000043345

**Published:** 2025-07-11

**Authors:** Zaliha Akkadin Candan, Ayşegül Aydin, Fatih Altan, Demet Unalan, Tülin Filik

**Affiliations:** a Ministry of Health Kayseri City Education and Research Hospital, Kayseri, Turkey; b Erciyes University Halil Bayraktar Health Services Vocational College, Kayseri, Türkiye; c Erciyes University Faculty of Dentistry, Kayseri, Türkiye.

**Keywords:** coping, earthquake, earthquake stress, trauma

## Abstract

The study aims to determine the trauma level of individuals who have experienced and witnessed the earthquake disaster and to reveal the strategies they used to cope with the stress of the earthquake. This cross-sectional and descriptive research population includes adults residing in Türkiye (n = 760). The IBM SPSS software was used to analyze the data, while *t* test and ANOVA tests were conducted for between-group comparison. Correlation analysis was conducted to evaluate the correlation between the variables. The study revealed that women, single individuals, associate degree graduates, those with income below minimum wage, residents of earthquake-affected cities, people who lost relatives in the earthquake, lived in non-earthquake-resistant houses, had active fault lines in their city, and lacked earthquake education had higher post-earthquake trauma levels. A negative correlation was found between age and trauma, while a positive correlation emerged between age and coping strategies. Religious coping was positively linked to emotive limitation and cognitive structure, while positive reappraisal and seeking social support were negatively associated with behavioral, emotive, cognitive, and sleep problems. These findings suggest that psychosocial support and education cannot overlook individual and geographical differences.

## 1. Introduction

According to the World Health Organization, an earthquake is “a violent and abrupt shaking of the ground, caused by movement between tectonic plates along a fault line in the earth’s crust.”^[1]^ Earthquakes, one of the most common disasters, affect individuals’ economic and social aspects and decrease their quality of life. In addition, financial losses, disabilities, and death are among the main difficulties experienced by individuals after an earthquake.^[2]^ Earthquakes that occurred worldwide between 1998 and 2017 caused the death of approximately 1 million people. The earthquake affected 125 million socially and economically on the specified dates.^[1]^

Because of its location, Türkiye has a geological structure with many tectonic movements. A 60% of loss of life and property resulting from natural disasters in the country are caused by earthquakes. On average, Türkiye experiences an earthquake that can be considered significant once every 5 years. On February 6th, 2023, the first earthquake, which was centered in Kahramanmaraş Pazarcik, occurred at 04.17 am with a magnitude of 7.7, and the second earthquake that occurred approximately 9 hours after it with a magnitude of 7.6 which was centered in Elbistan destroyed 11 cities, 62 districts, and 10,190 villages.^[3]^ In the heavily destroyed region, 53,537 people lost their lives, and 107,213 citizens were injured.^[4]^ The earthquake is considered among the deadliest earthquakes of the 21st century.^[5–8]^ Studies in the literature have revealed that individuals exposed to disasters such as earthquakes suffer from various health problems.^[9–11]^

It should be noted that post-traumatic stress disorder (PTSD) is the most common form of psychiatric morbidity after disasters.^[12,13]^ According to the American Psychiatric Association, PTSD is a psychiatric condition that may occur in people who have experienced or witnessed a traumatic event or series of traumatic events. Individuals often experience the event or events as emotionally or physically harmful or life-threatening. The symptoms of PTSD are examined in 4 main categories: intrusion, avoidance, changes in cognition and mood, changes in arousal and reactivity.^[14]^ Negative mental health impacts can persist for months or even years after an earthquake, potentially outlasting physical injuries caused by the disaster itself.^[15]^ Therefore, greater attention should be paid to monitoring and supporting post-disaster mental health.^[16]^ There are many studies in the literature investigating the prevalence and risk factors of PTSD after an earthquake. Studies have shown that the prevalence of PTSD varies between 14.5 and 48.2 and is higher in more affected regions than in less affected regions. It has been emphasized that the quality of life of individuals living in temporary housing is negatively affected.^[17]^ The earthquake victims whose buildings are damaged increase the risk of PTSD.^[18]^

The purpose of this study is to reveal the trauma level of individuals who experienced and witnessed the earthquake disaster and the strategies they use to cope with the stress of the earthquake. For this purpose, the research questions were determined as follows.

1. What are the socio-demographic characteristics of the participants and their earthquake-related situations (being in a disaster area, losing a loved one in a disaster, living on an active fault line, earthquake resistance of the house, receiving earthquake training)?
2. What are the trauma and stress levels of the participants after the earthquake?
3. What strategies did the participants use to cope with earthquake stress?
4. Is there a relationship between the socio-demographic characteristics of the participants and their trauma and stress levels after the earthquake?
5. Is there a relationship between the participants’ earthquake-related variables and their trauma and stress levels after the earthquake?
6. Is there a relationship between the participants’ earthquake-related variables and their strategies for coping with earthquake stress?
7. Is there a relationship between the participants’ socio-demographic characteristics and their strategies for coping with earthquake stress?
8. Is there a relationship between the participants’ post-earthquake trauma and stress levels and their strategies for coping with earthquake stress?

## 2. Method

### 2.1. Design, sample selection, and procedure

This study was conducted in order to determine the trauma level of individuals after the earthquake disaster centered in the city of Kahramanmaraş and affected 11 cities directly on February 06, 2023 and to reveal the relation between the trauma level and strategies used for coping with earthquake stress. The universe of the descriptive cross-sectional study conducted using the relational screening model consists of adults living in Turkey.

The research survey was prepared online and shared via various social media channels (Instagram, X, LinkedIn, WhatsApp, etc). The criteria for inclusion in the study were: being 18 years of age or older, being able to read and write, being able to use online technologies, being in settlements where the effects of the earthquake were felt, and agreeing to participate in the study voluntarily. Those with any physical or mental illness that could affect the study’s results were excluded. On the first page of the online survey, participants were informed about the duration and purpose of the study, and they were asked to fill out the mandatory informed consent form before accessing the survey. The “Personal Information Form,” which includes socio-demographic characteristics and their earthquake-related conditions, the “Strategies for Coping with Earthquake Scale,” and the “Post- Earthquake Trauma and Stress Level Identification Scale” were used as data collection tools.

### 2.2. Ethical considerations

The study was conducted according to the Declaration of Helsinki, with approval by the Ethical Committee of Erciyes University (March 20, 2023 dated and 2023/15 numbered approval) and informed consents of all participants were obtained.

### 2.3. Participants

The research sample consisted of 502 females (66.1%) and 258 (33.9%) males affected by the Kahramanmaraş earthquakes, aged between 18 and 73, with an average age of 33.3 ± 12.5. 50.8% (386) of the participants were single, 35.1% (267) were university graduates, and 35% (266) had an income twice the minimum wage. A 47.2% (359) of the participants were from the disaster area, and 42.1% (320) were from Central Anatolia, where the earthquake was felt most intensely. A 34.2% (260) of the participants reported that they had lost a relative in the earthquake, 77.4% (588) reported that there was an active fault line in the province they lived in, 49.6% (377) reported that their houses were earthquake resistant, and 64.6% (491) reported that they had not received earthquake training before (Table 1).

**Table 1:** Distribution of *PETSLIS* and *SCES* scores according to various variables.

Variables		n (%)	PETSLIS subscale					SCES subscale		
			BP	EL	AF	CS	SP	RC	PR	SSS
Gender	Famale	502 (66.1)	10.53	15.48	13.27	15.89	10.17	16.55	19.00	13.21
	Male	258 (33.9)	8.57	12.92	11.61	13.21	8.42	15.89	19.45	12.46
	*P*		.000**	.000**	.000**	.000**	.000**	.015*	.122	.004**
Marital Status	Single	386 (50.8)	10.29	15.18	12.95	15.52	9.93	15.90	18.91	12.34
	Married	374 (49.2)	9.43	14.03	12.45	14.42	9.21	16.77	19.40	13.61
	*P*		.007**	.007**	.059	.000**	.010*	.001**	.081	.000**
Educational	Primary-middle	35 (4.6)	8.06 ^ab^	12.77^ab^	12.89 ^ab^	14.29 ^ab^	8.74^ab^	17.23 ^ab^	20.03	12.57 ^ab^
	High school	105 (13.8)	10.03^ab^	15.18 ^ab^	12.71 ^ab^	15.10 ^ab^	9.35 ^ab^	16.56 ^ab^	19.03	12.40 ^ab^
	Associate degree	244 (32.1)	10.96^a^	15.60^a^	13.35^a^	16.17^a^	10.38 ^a^	16.71 ^a^	19.19	12.59^a^
	Undergraduate	267 (35.1)	9.09^b^	13.72^b^	12.31^b^	14.25 ^b^	9.15^b^	16.12 ^ab^	19.37	13.31 ^ab^
	Postgraduate	109 (14.3)	9.75^ab^	14.68 ^ab^	12.19 ^ab^	14.22 ^ab^	9.34 ^ab^	15.49 ^b^	18.41	13.63^b^
	*P*		.000**	.001**	.013*	.000**	.002**	.015*	.136	.009**
Income	Minimum wage↓	52 (6.8)	11.38^a^	15.54	12.88	16.02^a^	10.92^a^	16.48	19.60	11.36^a^
	Minimum wage	98 (12.9)	10.51 ^ab^	14.71	13.17	15.27 ^ab^	9.74^abc^	16.79	19.49	13.06^b^
	Minimum wage × 2	266 (35)	9.30^b^	14.01	12.47	14.24^b^	9.17^b^	16.30	19.42	13.68^b^
	Minimum wage × 3↑	60 (7.9)	9.28 ^ab^	13.97	11.87	14.62 ^ab^	9.00^c^	15.37	18.88	13.42^b^
	*P*		.003**	.297	.136	.026*	.015*	.100	.693	.000**
Geographical area	Central Anatolia	320 (42.1)	9.13 ^a^	14.04	12.22^a^	14.12 ^a^	9.17^abc^	16.29	19.18	13.33
	Aegean	11 (1.4)	9.18^abc^	14.73	12.91^abc^	14.73^abc^	8.27^abc^	14.91	18.36	11.82
	Mediterranean	104 (13.7)	11.15^b^	15.45	13.65^b^	16.17 ^b^	10.35 ^ab^	15.93	19.08	12.47
	Southeastern Anatolia	61 (8)	11.18 ^b^	16.06	14.38 ^c^	16.28 ^b^	10.51 ^ab^	17.23	19.54	13.16
	Black Sea	18 (2.4)	7.72 ^c^	13.89	12.00^abc^	12.39^abc^	7.28 ^c^	15.50	19.17	13.72
	Eastern Anatolia	224 (29.5)	10.25^bc^	14.93	12.56 ^ab^	15.63^ac^	9.87^abc^	16.49	19.13	12.60
	Marmara	22 (2.9)	9.00^abc^	12.41	12.77^abc^	13.91^abc^	8.77^abc^	16.09	18.68	13.09
	*P*		.000**	.051	.000**	.000**	.001**	.205	.959	.090
Living place	11 provinces affected by the earthquake	359 (47.2)	10.90	15.40	13.26	16.08	10.29	16.58	19.18	12.63
	Other Provinces	401 (52.8)	8.94	13.92	12.22	13.99	8.94	16.11	19.13	13.26
	*P*		.000**	.000**	.000**	.000**	.000**	.073	.852	.011*
Losing a relative in an earthquake	Yes	260 (34.2)	11.13	16.18	13.82	16.17	10.57	16.55	18.93	12.68
	No	500 (65.8)	9.21	13.81	12.13	14.36	9.06	16.22	19.27	13.11
	*P*		.000**	.000**	.000**	.000**	.000**	.215	.204	.245
Active fault line status in the province of residence	Yes	588 (77.4)	10.31	14.88	12.83	15.36	9.88	16.41	19.08	12.78
	No	172 (22.6)	8.36	13.73	12.30	13.69	8.55	16.07	19.42	13.60
	*P*		.000**	.019*	.093	.000**	.000**	.283	.304	.005**
Earthquake resistance of the house	Yes	377 (49.6)	9.12^a^	13.74^a^	12.26^a^	14.42^a^	9.01 ^a^	16.34	19.51^a^	13.44^a^
	No	86 (11.3)	12.35^b^	17.65^b^	14.74^b^	17.12^b^	11.28^b^	16.86	18.16^b^	11.49^b^
	I don’t know	297 (39.1)	10.10^c^	14.85^c^	12.69^a^	15.08^a^	9.81^c^	16.17	18.99^ab^	12.79^c^
	*P*		.000**	.000**	.000**	.000**	.000**	.288	.007**	.000**
Getting earthquake training	Yes	269 (35.4)	9.37	13.79	12.42	14.41	9.27	15.89	18.97	12.99
	No	491 (64.6)	10.14	15.07	12.87	15.30	9.75	16.57	19.26	12.95
	*P*		.021*	.004**	.102	.006**	.100	.012*	.327	.884

a, b, and c = there is no significant difference in groups containing the same letters.

AF = affective, BP = behavior problems, CS = cognitive structure, EL = emotive limitation, PETSLIS = Post- Earthquake Trauma and Stress Level Identification Scale, PR = positive reappraisal, RC = religious coping, SCES = Strategies for Coping with Earthquake Scale, SP = sleep problems, SSS = seeking social support.

* *P* < .05.

** *P* < .01.

### 2.4. Measures

*Personal information form:* there are 11 questions in the personal information form designed by the researchers based on literatüre.^[19,20]^ The variables collected include socio-demographic characteristics (age, gender, marital status, education and income status, region of residence), as well as earthquake-related variables such as being in 11 provinces that were disaster areas in the earthquake (Adana, Adiyaman, Diyarbakir, Elaziğ, Gaziantep, Hatay, Kahramanmaraş, Kilis, Malatya, Osmaniye, Şanliurfa), losing a relative in the earthquake, living on an active fault line, earthquake resistance of the house, and receiving earthquake training.

*Strategies for Coping with Earthquake Scale (SCES*): the scale, which consists of 16 questions and 3 sub-dimensions developed by Yöndem and Eren, is a 4-point Likert (Never, …, Always) type.^[21]^ The scale consists of the most frequently used sub-dimensions against earthquake stress: “religious coping (RC)” (5 items, 5–20 points), “positive reappraisal (PR)” (6 items, 6–24 points), and “seeking social support (SSS)” (5 items, 5–20 points). Items 4 (I try to keep my feelings to myself) and 12 (I prefer not to talk about my fears and anxieties) in the seeking social support dimension are scored in reverse because they indicate that the individual does not seek social support. The total score is not calculated in the scale. A high score for each dimension reflects that the individual uses the coping strategy more, while a low score reflects that the individual uses it less. For the current study, the Cronbach alpha reliability coefficients for the scale sub-dimensions were determined as 0.81 for RC, 0.80 for PR, and 0.69 for SSS.

*Post-Earthquake Trauma and Stress Level Identification Scale (PETSLIS):* the scale developed by Tanhan and Kayri aims to measure traumatic symptoms that may occur in individuals after an earthquake.^[22]^ The scale consists of 20 questions on a five-point Likert (strongly disagree, …, strongly agree) scale. Eighteen scale items have positive expressions, and 2 (items 11 and 12) have negative expressions. The lowest score that can be obtained from the scale is 20, and the highest score is 100. Increasing scores on the scale indicate an increase in exposure to the earthquake. The scale has 5 sub-dimensions: “behavioral problems (BP)” (4 items/α = 0.774), “emotional limitation (EL)” (4 items/α = 0.847), “affective (AF)” (4 items/α = 0.601), “cognitive structure (CS)” (4 items/α = 0.793), and “sleep problems (SP)” (3 items/α = 0.830). The Cronbach alpha reliability coefficient of the total scale for the current study was determined to be 0.92.

### 2.5. Statistical analysis

IBM SPSS software was used for data analysis, while *t* test and ANOVA test were conducted and post hoc tukey method was used for between-groups comparison. Pearson correlation analysis was conducted to determine the relations between the variables. Significance level of the data was accepted as *P* < .05.

## 3. Findings

The research data were collected from 51 provinces, 11 of which were disaster areas, 47.2% of which were from all regions of our country after the earthquakes centered in Kahramanmaraş. The demographic and socio-cultural characteristics of the participants are given in Table 1.

In our research, it was discovered that all PETSLIS subscale scores and SCES RC and SSS subscale scores of women were significantly higher compared to men, and single participants’ PETSLIS BP, EL, CS and SP subscale scores and SCES RC, and SSS subscale were significantly higher compared to married participants (Table 1).

When PETSLIS subscale scores were analyzed in terms of the participants’ level of education, statistically meaningful differences were observed in all subscales, and the difference between groups was seen to arise from that associate degree graduates had higher scores than high school graduates. When SCES scores were analyzed in terms of the level of education, it was determined that the statistically significant difference in RC and SSS subscales arose from the participants having associate and postgraduate degrees (Table 1).

When subscale scores were analyzed in terms of income level, the participants having an income lower than the minimum wage were observed to have significantly higher scores in CS, AF, BP, and SP subscales compared to those with an income twice the minimum wage, and significantly lower scores in the SSS subscale compared to other groups (Table 1).

When PETSLIS subscale scores were analyzed in term of the geographical locations of the participants, statistically significant differences in the BP, AF, SC, and SP subscale scores were observed, and the differences arose from the high scores of the participants from Mediterranean, Southeastern Anatolia, and Eastern Anatolia regions (Table 1).

All PETSLIS subscale scores of the participants residing in the 11 cities that were affected by the Kahramanmaraş centered earthquake, having deceased relatives and stating that their houses were non-earthquake resistant were observed to be significantly higher than the other participants. Additionally, the SCES SSS subscale scores of the participants residing in one of the 11 cities affected by the earthquake were found to be significantly lower compared to those living in other cities. The SCES PR and SSS subscale scores of those living in earthquake-resistant houses were found to be significantly higher compared to others (Table 1).

It was determined that the participants who stated that the city they resided in had an active fault line had significantly higher PETSLIS BP, EL, CS, and SP subscale scores and significantly lower SCES SSS subscale scores compared to the participants residing in areas with no active fault lines (Table 1).

When subscale scores were analyzed in terms of the participants receiving earthquake training, PETSLIS BP, EL, and CS trauma levels and earthquake stress, and RC subscale scores of those who did not receive training were statistically significantly higher compared to those who received an education (Table 1).

When Cronbach alpha internal consistency coefficients were analyzed related to the subscales presented in Table 2, it was observed that the scales were quite reliable. The Cronbach alpha coefficient was determined as 0.925 for the whole PETSLIS and α = 0.802 for the whole SCES, and the scales were quite reliable. It was found that the participants had the highest scores in SP subscale and lowest scores in the BPs subscale in the post-earthquake trauma level identification scale subscales while they had the highest scores in PR in the strategies for coping with earthquake scale subscales.

**Table 2:** Descriptive information and correlation analysis results regarding age variable, PETSLIS, and SCES sub-dimensions.

	α	MEAN	SD	1	2.	3.	4.	5.	6.	7.	8.	9.
1. Age	–	33.29	12.48	1								
2. BP	0.774	9.58	3.79	-0.148**	1							
3. EL	0.847	9.87	4.38	-0.148**	0.638**	1						
4. AF	0.601	14.62	5.88	-0.092*	0.573**	0.577**	1					
5. CS	0.793	12.71	3.67	-0.198**	0.671**	0.676**	0.639**	1				
6. SP	0.830	14.98	4.28	-0.116**	0.678**	0.508**	0.501**	0.658**	1			
7. RC	0.815	16.33	3.56	0.075*	0.041	0.080*	0.167**	0.097**	0.016	1		
8. PR	0.805	19.16	3.80	0.143**	-0.132**	-0.221**	0.044	-0.073*	-0.098**	0.324**	1	
9. SSS	0.686	12.96	3.39	0.172**	-0.094**	-0.093*	0.061	-0.010	-0.080*	0.149**	0.227**	1

AF = affective, BP = behavior problems, CS = cognitive structure, EL = emotive limitation, PETSLIS = Post- Earthquake Trauma and Stress Level Identification Scale, PR = positive reappraisal, RC = religious coping, SCES = Strategies for Coping with Earthquake Scale, SP = sleep problems, SSS = seeking social support.

* *P* < .05.

** *P* < .01.

When Table 2 was analyzed, it was determined that the age average of the participants was 33.29 ± 12.5 and the participants’ age had negative significant relations with the PETSLIS subscale scores and positive significant relations with the SCES subscale scores. According to these findings, it can be said that as the participants’ ages increase, their earthquake-related traumas related to behavioral, emotional, AF, cognitive, and SP decrease; and they use PR, social support seeking and RC strategies more to cope with earthquake stress.

When the relations between PETSLIS and SCES were analyzed, a low and positive significant relation was found between the RC subscale scores and EL, AF, and CS subscale scores, and low negative significant relation between PR subscale scores and BP, EL, CS, and SP subscale scores, and low negative relation between SSS subscale scores and BP, EL, and SP subscale scores.

## 4. Discussion

It was determined in the study that the participants had the highest scores in the “SP” subscale and the lowest scores in the “BPs” subscale among the post-earthquake trauma and stress level identification subscales. It is understood that sleep disturbances are the most sensitive physiological dimension in the context of the physiological effects of trauma. Sleep is the physiological process affected the most by any mental disease and distress from life events (this could also be a pleasant event). Sleep disturbance also affects other psychological factors and coping negatively since sleep is a process recurring every day that renews and restores the person mentally and physically. It is of great importance in human life.

On the other hand, BPs were observed at the lowest rate in the participants in our study. BPs can be regarded as an indication of a relatively more severe picture of psychopathology, and the occurrence of BPs indicates psychosis, which refers to the weakening of reality evaluation and impulse control. As a result of a study conducted by Çelik, similar findings were found in this study.^[23]^ As a result of the research conducted by Kurt and Gülbahçe examining the PTSD of those who experienced the earthquake, it was determined that 42.6% of the participants had mild traumatic stress symptom levels, 36.7% were moderate, 19.4% were moderate-severe, and 1.3% were severe.^[24]^ In the research conducted by Sönmez on the psychological effects of the earthquake, psychological support, and coping with fear, it was emphasized that individuals may experience anxiety, depression, physical symptoms, dissociative symptoms, sexual function, and sleep disorders after the earthquake.^[25]^

Among the strategies for coping with the earthquake stress scale, the participants had the highest score in the PR subscale. Positive reappraisal is quite important for coping, and it refers to evaluating the new situation realistically and trying to adapt to the new situation in this context. It is expected to be a negative relation between PR subscale scores. BP, EL, CS, and sleep problem subscale scores, and the processes of both problem solving and the adaptation to the new situation will occur through PR, which will positively affect behavioral, emotive, CS, and sleep physiology. In the study conducted after the Elaziğ earthquake researching the early psychological effects of post-earthquake trauma and possible risk factors, a quite highly positive relation were discovered between the depression scale and anxiety scale, a moderate positive relation between peritraumatic dissociation scale and anxiety and depression scales and a moderate positive relation between the PTSD Control List and all the other scales implemented.^[26]^

It can be claimed that as the ages of the participants increase, the earthquake-related traumas related to behavioral, emotive, cognitive, and SP decrease, and the participants use PR, seeking social support, and RC strategies more frequently to cope with the stress of the earthquake.

Among the common risk factors specified for post-earthquake psychological problems, the female gender plays a role in explaining the critical and chronic nature of PTSD.^[27–29]^ In this study, women’s scores were significantly higher compared to men’s in all of the post-earthquake trauma and stress level identification subscales, and in strategies for coping with earthquake scale, RC, and social support seeking subscales. Study findings evaluate similar cases that exist in the literature. The gender variable was determined to be a significant predictor.^[23,30]^ As a result of the research conducted by Nearchou and Douglas, it was revealed that traumatic stress decreases when hope increases, and psychological resilience has a mediating role in the relationship between traumatic stress and depression.^[31]^ The study by Neumayer and Plümper emphasized that women are more affected by natural disasters than men.^[32]^ A study conducted by Kardaş and Tanhan determined that the gender variable was one of the significant predictors.^[33]^ Another study revealed that earthquakes significantly affect women’s well-being and therefore have a broader impact on the entire family.^[11]^

In this study, single participants’ scores in BPs, EL, CS, and SP subscales of PETSLIS, and RC and seeking social subscales of SCES were observed to be significantly higher than the scores of those who are married. Being married, being the head of a family, being unemployed, having a low income, and living in temporary housing carries a higher risk of PTSD.^[34–36]^ Aslam and Tariq indicate that married survivors tend to experience greater amounts of trauma, depression, and stress, while Musa et al show that trauma, depression, and stress occur more frequently in single survivors.^[37,38]^

Associate degree graduates scored significantly higher than high school graduates in the PETSLIS subscales, and associate degree graduates and high school graduates scored significantly higher than the other groups in SCES RC and SSS subscales. Brewin et al, who conducted a meta-analysis on the risk factors of PTSD in adults who were exposed to trauma, found that the educational background factor did not have a significant relation.^[34]^

Participants with an income lower than the minimum wage were observed to have significantly higher scores in the CS, BPs, and SP subscales compared to those with an income twice the minimum wage, and significantly lower scores in the seeking social support subscale compared to the other groups. In the studies conducted after the disasters in the literature, the possibility of defenseless individuals visiting any expert is observed to be low due to the insufficiency of financial sources and fear of stigmatization, it is also observed that individuals with low income who have history of trauma are more likely to consult primary healthcare rather than mental health professionals.^[29,36,39,40]^

Participants from the Mediterranean, Southeastern Anatolia, and Eastern Anatolia regions were observed to have significantly high scores in PETSLIS BP, AF, CS, and SP subscales.

All PETSLIS subscale scores of the participants who lived in one of the 11 cities affected by the Kahramanmaraş-centered earthquake, lost a relative in the earthquake, and stated that the house they resided in was not earthquake-resistant were observed to be significantly higher than the others. In the study by Bilgili and Bolat, the participants stated that they experienced intense emotions such as losing their relatives and houses, and the uncertainty of the future.^[41]^ Earthquakes are primarily associated with psychological issues such as depression, anxiety, stress, and PTSD. These anxiety and stress disorders can reduce the resilience levels of earthquake survivors.^[42]^ A study conducted by Xi et al on earthquake survivors found high levels of depression, anxiety, and PTSD symptoms in this group.^[43]^

The SCES SSS subscale scores of participants residing in the 11 cities affected by the earthquake were significantly lower than those of others.

The SCES Positive Reappraisal and SSS subscale scores of the participants who stated that the earthquake-resistant house they resided in was significantly higher than others.

The study found a low level of positive relation between the RC subscale scores and EL, AF, and CS subscale scores. In a study conducted with individuals in Pakistan who survived the earthquake, it was found that having social capital and a religious disposition had a protective, buffering effect against PTSD and increased resistance.^[35]^

In the study, a low level of a significant negative relation was observed between seeking social support subscale scores and BP, EL, and SP subscale scores. This result was expected because seeking social support is an effort to resolve all kinds of negativity and positively affects behavioral, AF, and cognitive states and sleep physiology. In the research of Cofini et al, participants with PTSD symptoms frequently used incompatible coping strategies. They scored highest in the denial, acting out, separating behaviorally, and self-blame categories.^[44]^ Sharma and Kar stated that adolescents used various coping strategies such as talking to others, praying, helping others, and hoping for the best in coping with post-traumatic stress and depression after the earthquake.^[45]^ Also, a study by Zhou et al found that self-esteem and hope mediate the relations between social support, PTSD, and growth in adolescents after the earthquake.^[19]^ In a study conducted by Alipour and Ahmadi, victims reported that social support after the trauma experienced can prevent PTSD.^[46]^ Studies have reported that social support can be a coping method that earthquake victims can use against negative psychopathology they may experience and emphasized that social interactions are protective against stress and depression.^[19]^ Another study has revealed that earthquake victims with a high perception of social support increase their quality of life and resilience.^[47]^

Consequently, every major disaster requires retrospective research. This way, it can be learned what emergency medical services and long-term care require, and how to improve them. The findings of this study are similar to those of the studies in the literature.

## 5. Conclusion

This research approaches the trauma and stress levels and strategies for coping with earthquake stress of individuals after the Kahramanmaraş-centered earthquakes that occurred on February 06, 2023 in the context of demographic, socio-cultural, and geographical factors. The findings show that individuals’ trauma levels and strategies for coping with earthquake stress are significantly affected by various factors.

Women are more sensitive to trauma compared to men, and they use RC and seeking social support strategies more. Single individuals have both higher trauma and stress levels and turn to RC more compared to married individuals. Individuals with low income have higher trauma levels but fewer tendencies for seeking social support compared to others. As the education level increases, trauma levels decrease, and seeking social support strategies is used more frequently. Those who live on active fault lines and have lost a relative in the earthquake experience trauma effects, such as behavioral, cognitive, and SP, more intensely. Individuals who received earthquake training are observed to have lower trauma levels and use strategies such as PR and seeking social support more compared to others. As age increases, traumatic earthquake-related effects lessen, and the tendency for positive coping strategies increases.

These results reveal the importance of considering personal and regional differences in psychosocial support processes and training programs. It is recommended that special intervention programs be developed especially for individuals residing in areas affected by the earthquake, such as low-income groups, women, and single individuals. In addition, it is emphasized that increasing earthquake preparation and endurance training is critical in individual and social trauma management.

## 6. Recommendations

Based on the results of this study, the following recommendations for future studies are given below.

- Target group interventions: special psychosocial support programs should be developed for women, singles, and low-income individuals.
- Support appropriate to local needs: awareness and support activities should be increased for those in regions heavily affected by the earthquake.
- Dissemination of earthquake education: earthquake education, including psychological preparation, should be delivered to all segments of society.
- Gender sensitive approach: appropriate support systems should be created, considering the different coping methods of women and men.
- Age-appropriate support programs: programs that support stress management for the young and positive coping for the elderly should be implemented.
- Long-term follow-up studies: studies should be conducted to examine the long-term effects of trauma.
- Community-based support: supportive community programs should be implemented at the neighborhood level with the participation of local actors.

## 7. Limitations

The most important limitation of this research is the sampling method. It is thought that providing a certain number of participants from the earthquake affected and unaffected provinces by conducting stratified sampling will take the research to a further level.

## Author contributions

**Conceptualization:** Zaliha Akkadin Candan, Ayşegül Aydin, Fatih Altan, Demet Unalan.

**Data curation:** Ayşegül Aydin.

**Formal analysis:** Ayşegül Aydin.

**Methodology:** Ayşegül Aydin, Fatih Altan, Demet Unalan.

**Project administration:** Ayşegül Aydin.

**Writing – original draft:** Zaliha Akkadin Candan, Ayşegül Aydin, Fatih Altan, Demet Unalan, Tülin Filik.

**Writing – review & editing:** Ayşegül Aydin, Fatih Altan.
